# Estimating asymptomatic SARS-CoV-2 infections in a geographic area of low disease incidence

**DOI:** 10.1186/s12879-021-06054-2

**Published:** 2021-04-15

**Authors:** Valeria Caturano, Barbara Manti, Fortunata Carbone, Vito Alessandro Lasorsa, Roberta Colicchio, Mario Capasso, Antonio Leonardi, Giuseppe Matarese, Tommaso Russo, Paola Salvatore

**Affiliations:** 1 Department of Molecular Medicine and Medical Biotechnology, University of Napoli Federico II, Via S. Pansini 5, 80131 Naples, Italy; 2 Istituto di Endocrinologia e Oncologia Sperimentale, Consiglio Nazionale Delle Ricerche (IEOS-CNR), Naples, Italy; 3 CEINGE Biotecnologie Avanzate s.c.ar.l., Naples, Italy

**Keywords:** SARS-CoV-2, Covid-19, IgG, Disease prevalence, Asymptomatic individuals

## Abstract

**Background:**

The SARS-CoV-2 infection has emerged as a rapidly spreading infection. Today it is relatively easy to isolate Covid-19 symptomatic cases, while remains problematic to control the disease spread by infected but symptom-free individuals. The control of this possible path of contagion requires drastic measures of social distancing, which imply the suspension of most activities and generate economic and social issues. This study is aimed at estimating the percentage of asymptomatic SARS-CoV-2 infection in a geographic area with relatively low incidence of Covid-19.

**Methods:**

Blood serum samples from 388 healthy volunteers were analyzed for the presence of anti-SARS-CoV-2 IgG by using an ELISA assay based on recombinant viral nucleocapsid protein.

**Results:**

We found that 7 out of 388 healthy volunteers, who declared no symptoms of Covid-19, like fever, cough, fatigue etc., in the preceding 5 months, have bona fide serum anti-SARS-CoV-2 IgG, that is 1.8% of the asymptomatic population (95% confidence interval: 0.69–2.91%).

**Conclusions:**

The estimated range of asymptomatic individuals with anti-SARS-CoV-2 IgG should be between 26,565 and 112, 350. In the same geographic area, there are 4665 symptomatic diagnosed cases.

**Supplementary Information:**

The online version contains supplementary material available at 10.1186/s12879-021-06054-2.

## Introduction

Understanding the prevalence of asymptomatic individuals infected by SARS-CoV-2 is of crucial relevance, mainly due to the resurgence of pandemic that is occurring in the current autumn-winter season. Indeed, while it is relatively easy to isolate Covid-19 symptomatic cases, preventing them from infecting other individuals, it is problematic to control the spread of the disease by infected but symptom-free individuals [1–3]. The control of this possible path of contagion requires drastic measures of social distancing, which imply the suspension of most activities and generate economic and social issues.

The data on the number of asymptomatic infected subjects are not yet conclusive, also because the risk of exposure to the virus of the analyzed cohorts were different in different contests. In the case of a confined population, including many symptomatic cases, such as that of the cruise ship Diamond Princess, the proportion of asymptomatic or paucisymptomatic subjects among those positive for the presence of viruses in the nasopharyngeal swab was calculated to be 17.9% (95% CrI: 15.5–20.2%) [4]. In another population, that of Japanese citizens evacuated from Wuhan, potentially highly exposed to the infection, the proportion of asymptomatic subjects compared to all positive subjects for the presence of the virus was 33.3% (95% confidence interval: 8.3–58.3%) [5].

The lockdown had a clear effect on the infection spread [6, 7]. In particular, there many areas where the contagion was drastically limited. In Italy for example, there are regions, like Lombardy, where the number of infected subjects was high (93,761 on June 30, 2020; data from Italian Ministry of Health), while in other Regions of the Country, like in Campania, the number of infected subjects was significantly lower (4665), probably at least in part as a consequence of the imposed limitation of moving among Regions. In the “low-incidence” Regions the number of asymptomatic infected subjects is unknown and it cannot be excluded that the low diffusion of the contagion has also influenced the proportion of asymptomatic vs symptomatic individuals.

To address this point, we estimated the size of the asymptomatic infected population in a “low-incidence” Region of Italy (Campania Region, South Western Italy), by measuring anti-SARS-CoV-2 IgG in serum samples from a cohort of asymptomatic subjects.

In addition, it is known that studies on blood donor cohorts are useful to evaluate the prevalence, incidence and natural course of viral infection in the general population and may help to evaluate the Covid-19 outbreak [8].

## Materials and methods

We have randomly recruited 388 healthy volunteers (HV) aged between 19 and 68 years, shortly before the end of the lockdown period in Italy. The HVs declared that they had none of the symptoms frequently associated with the infection, such as fever, cough, fatigue etc., in the past five months. They had stayed in the Campania Region at least since December 1, 2019. We also examined serum samples from 13 symptomatic patients (SP), 7 of which hospitalized in the University Hospital Federico II.

Anti-SARS-CoV-2 IgG were measured with an ELISA assay, by commercially available kit (NovaTec GmbH, Germany), according to the manufacturer’s instruction (NovaLisa SARS-CoV-2 IgG) on fresh serum samples. ELISA is based on the detection of serum IgG directed against SARS-CoV-2 nucleocapsid recombinant antigen. The results of the assay are reported as the absorbance value at 450 nm × 10/cut-off control value. According to manufacturer instructions for the SARS-CoV-2 IgG assay value of < 9 was considered negative, 9 to 11 borderline/doubt and > 11 positive. The NovaLisa SARS-CoV-2 IgG test used has a sensitivity and specificity of 94.9% (95% confidence interval [CI]: 83.1–98.6%) and 96.2% (95% CI: 89.4–98.7%) respectively, as previously reported [9] and in agreement with the manufacturer.

## Results and discussion

**Fig. 1:**
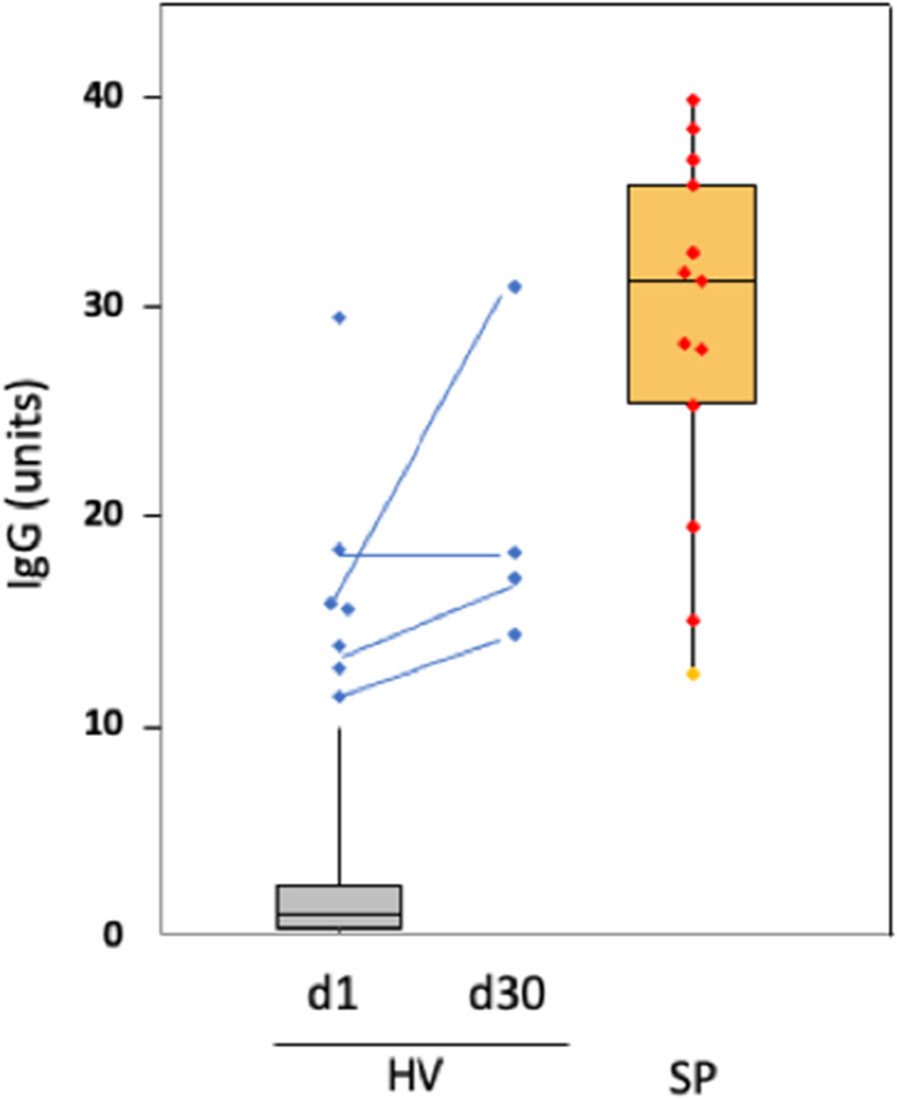
Serum SARS-CoV-2 IgG in asymptomatic healthy volunteers (HV, blue dots) and symptomatic patients (SP, red dots). In four HV, IgG were measured at day 1 (d1) and day 30 (d30). Box plot of HV d1 refers to 381 subjects with SARS-CoV-2 IgG levels below the positivity threshold. The yellow dot indicates the IgG levels in a paucisymptomatic patient (only showing anosmia) that had been positive for the presence of SARS-CoV-2 in nasopharyngeal swab, but was negative at the moment of blood sampling. The difference between the HV IgG values above 11 Units (blue dots) and HV IgG values below the threshold was significant (*p* = 7.80853*10^− 6^). The difference between the HV IgG values above 11 Units and SP is less significant (*p* = 0.002063)

The results showed that 7 out of 388 samples from HV have values higher that 11 Units (Fig. 1), that is the positivity threshold value indicated by the Manufacturer. In 4/4 subjects with positive values, a second serum sample, drawn 30 days after the previous one, confirmed the positivity. Positive subjects declared that they were unaware of the infection source. All the 13 SP had values higher than 11 Units.

We also performed an unbiased calculation of the changepoint(s) in the entire list of ELISA results (including HV, SP and repeated assays, *n* = 405), by using the R package Changepoint [10]. In brief, we searched data values (changepoints) where the statistical properties of the data before and after this value differed. To detect possible multiple changepoints, we used the Pruned Exact Linear Time (PELT) algorithm that required an interval of penalty values as the costs to segment the data in new groups. The PELT algorithm can create more groups (and changepoints) when lower penalty values are considered. With the “one changepoint” option (penalty 1951.07) the boundary value was 15.028, while with the “two changepoint” option (penalty 727.86) the threshold values were 7.911 and 25.193 (Supplementary Fig. 1). These two changepoints identified: i) a group of low values, ii) a group of high values and iii) a group of intermediate IgG values. By using these three groups, 376/388 HV are in the low range. The remaining 13 values are distributed in the other two groups, with one value in the high-level range. The values of 10 SP were in the high range and three of them in the intermediate range.

In conclusion, if we consider the positivity threshold indicated by the Vendor, the percentage of asymptomatic subjects with bona-fide anti-SARS-CoV-2 IgG is of (7/388) 1.8 (95% confidence interval: 0.69–2.91%). The percentages of HV positivity calculated on the basis of the changepoint analysis are: (1/388) 0.25% in the high-level range (95% confidence interval: 0–0.66%) and (12/388) 3.09% in the intermediate-level range (95% confidence interval: 1.65–4.53%). In agreement with previously data on the seroprevalance calculated on a larger southern Italian cohort with a percentage of positivity of 5.6% [11] closely related to our data.

However, although using the cut-off established by the Vendor the number of false positives and false negatives is very low for the NovaLisa SARS-CoV-2 IgG assay, it is not possible to exclude that false positive or false negative results could occur in the population screened in the present study [9]. On the other hand, due to these patients are asymptomatic and thus possibly removed from an active infection which would give a positive molecular assay, the confirmation of a positive ELISA result with a molecular test may not be viable.

On July 7, 2020, in the Campania region, 4747 cases of positivity for the presence of the virus in nasopharyngeal swabs have been ascertained on 145,538 subjects examined. Given that social distancing rules have been imposed since March 9, 2020, in accordance with Italian government decisions, and that the first Covid-19 cases in Campania were diagnosed on March the 2nd, it is reasonable that the healthy volunteers we recruited have been exposed to possible contagion without restrictions for a few weeks.

Considering that the population of the examined age range in the Campania Region is of about 3.85 million of people, the estimated range of asymptomatic individuals with anti-SARS-CoV-2 IgG should be between 26,565 and 112,350 that are much more than the 4665 symptomatic diagnosed cases. This study suggests that in a “low-incidence” Region, in a randomized population, the number of asymptomatic infected subjects is unknown and it cannot be excluded that the contagion diffusion is influenced by the proportion of asymptomatic vs symptomatic individuals. The incidence of SARS-CoV-2 infection could be higher than in the recorded cases, highlighting the importance of a massive population screening. Although molecular assays remain the reference method for identifying active infection, as the SARS-CoV-2 pandemic continues to spread in all over the word, today serological testing has become essential to understand the evolution of pandemic and to estimate its future [12, 13].

## Supplementary Information

**Additional file 1: Supplementary Figure 1** The changepoints were calculated by using the R function cpt.mean. PELT algorithm was used with the penalty method CROPS (Changepoints for a Range of Penalties) [10] [Killick & Eckley, 2014]. The penalties were calculated within the range: 3*log(*n*) < penalty value < 1000*log(*n*); *n*=405. Changepoints were at 7.911 and 25.193. P was calculated by Mann-Whitney Test.

## Data Availability

The data for this study are available from the corresponding authors on reasonable request.

## Abbreviations

Covid-19: COronaVIrus Disease 19
ELISA: Enzyme-linked immunosorbent assay
HV: Healthy volunteers
IgG: Immunoglobulin G
PELT: Pruned Exact Linear Time
SARS-CoV-2: Severe acute respiratory syndrome coronavirus 2
SP: Symptomatic patients
