# Supplementary material for: Estimating asymptomatic SARS-CoV-2 infections in a geographic area of low disease incidence
Source: BMC Infect Dis. 2021 Apr 15;21:350. doi: 10.1186/s12879-021-06054-2 (PMC8046491; doi:10.1186/s12879-021-06054-2)
Supplement: Supplementary file 1 — Additional file 1: Supplementary Figure 1 The changepoints were calculated by using the R function cpt.mean. PELT algorithm was used with the penalty method CROPS (Changepoints for a Range of Penalties) [10] [Killick & Eckley, 2014]. The penalties were calculated within the range: 3*log(n) < penalty value < 1000*log(n); n=405. Changepoints were at 7.911 and 25.193. P was calculated by Mann-Whitney Test. [file 12879_2021_6054_MOESM1_ESM.docx]

**Estimating asymptomatic SARS-CoV-2 infections in a geographic area of low disease incidence**

Valeria Caturano^1#^, Barbara Manti^1#^, Fortunata Carbone^2^, Vito Alessandro Lasorsa^1,3^, Roberta Colicchio^1^, Mario Capasso^1,3^, Antonio Leonardi^1^, Giuseppe Matarese^1,2^, Tommaso Russo^1,^*, Paola Salvatore^1,3,^*

^1^Department of Molecular Medicine and Medical Biotechnology, University of Napoli Federico II, Naples, Italy; ^2^Istituto di Endocrinologia e Oncologia Sperimentale, Consiglio Nazionale Delle Ricerche (IEOS-CNR), Naples, Italy; ^3^CEINGE Biotecnologie Avanzate s.c.ar.l. Napoli, Italy.

^#^These authors equally contributed.

**Supporting Information**


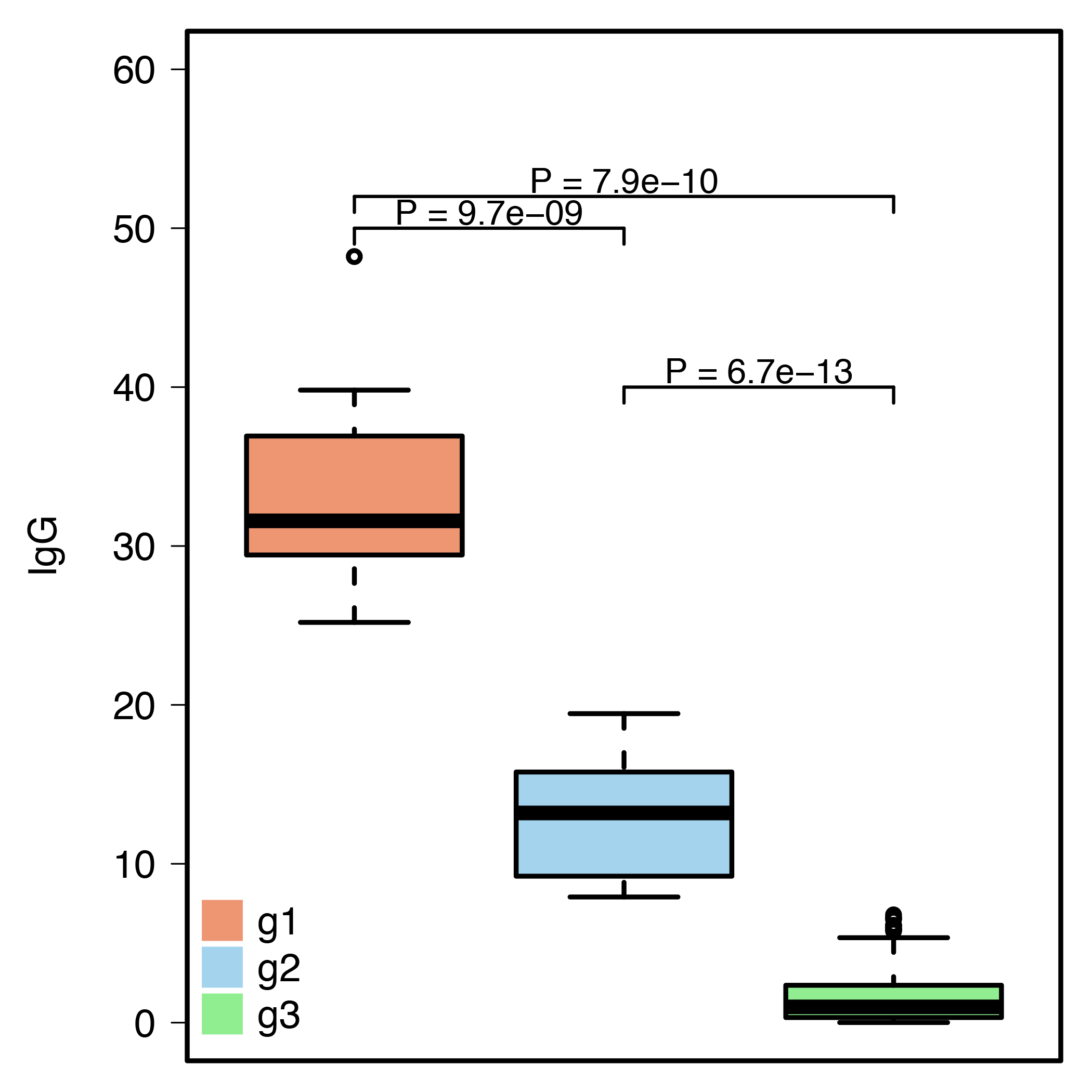


**Supplementary Fig 1** The changepoints were calculated by using the R function cpt.mean. PELT algorithm was used with the penalty method CROPS (Changepoints for a Range of Penalties) [Killick & Eckley, 2014]. The penalties were calculated within the range: 3*log(n) < penalty value < 1000*log(n); n=405. Changepoints were at 7.911 and 25.193. P was calculated by Mann-Whitney Test.
